# Proton pump inhibitors in systemic sclerosis: should we exercise caution? Insights from a large-scale data analysis

**DOI:** 10.1007/s10067-025-07686-4

**Published:** 2025-09-12

**Authors:** Irakli Tskhakaia, Diego Lema, Nanuka Tsibadze, Justin Riley Lam, Apoorva Subramanian, Arthur Lau

**Affiliations:** 1 Department of Medicine, Jefferson Einstein Hospital, 5501 Old York Road, Philadelphia, PA 19141 USA; 2 Division of Rheumatology, Jefferson Einstein Hospital, Philadelphia, PA USA

**Keywords:** Proton Pump Inhibitors (PPIs), Systemic Sclerosis (SSc)/Scleroderma, Chronic Kidney Disease (CKD), Osteoporosis, Neurodegeneration/Alzheimer’s Disease/Vascular Dementia, Drug Safety/Long-term Adverse Effects

## Abstract

**Background:**

Systemic sclerosis (SSc) is a chronic autoimmune disorder often accompanied by gastroesophageal reflux disease (GERD), necessitating frequent use of proton pump inhibitors (PPIs). While PPIs mitigate GERD symptoms and protect against lung injury, concerns about their long-term safety, particularly regarding chronic kidney disease (CKD), osteoporosis, and Alzheimer’s disease, are growing. This study aimed to assess whether the adverse effects of PPI are amplified in patients with scleroderma.

**Methods:**

This was a retrospective observational analysis that utilized the TriNetX research network, including over 130 million patients globally. The study population comprised 6800 SSc patients on PPIs and 1,889,433 GERD patients on PPIs. Outcomes were evaluated pre- and post-propensity score matching for demographic and clinical factors. Risks of CKD (including stages 3, 4, 5, ESRD, hemodialysis dependence), osteoporosis, vascular dementia, and Alzheimer’s disease were assessed.

**Results:**

The SSc cohort exhibited higher risks of developing CKD (attributable risk [AR] 2.8%, *p* < 0.01) and osteoporosis (AR 9%, *p* < 0.01) after matching, compared to the GERD cohort. Notably, CKD stages 4 and 5 showed minimal differences between groups. SSc patients on PPI had lower risks of Alzheimer’s disease (AR − 0.7%, *p* < 0.01). While the findings highlight an amplified risk of CKD and osteoporosis in SSc patients, the differences in advanced renal disease were modest.

**Conclusion:**

PPI therapy remains indispensable in SSc management. While potentially associated with a slight but significant increase in the risks of CKD and osteoporosis, these adverse effects do not negate the critical role of PPIs in mitigating GERD and its serious pulmonary complications. A strategy of targeted monitoring is recommended to maximize safety.

**Table:** 

**Key Points** • *Identified Amplified Risks in SSc: This large-scale study reveals that SSc patients on PPIs face significantly higher risks of chronic kidney disease and osteoporosis compared to GERD patients on PPIs, suggesting SSc pathophysiology amplifies PPI-associated adverse effects.* • *Uncovered Neuroprotective Association: Contrary to general population trends, SSc patients on PPIs showed a reduced risk of Alzheimer’s disease, potentially linked to immunosuppressant use, closer monitoring, or unique disease mechanisms—a finding warranting further investigation.* • *Provided Real-World Safety Evidence: Leveraging global data (> 130 million patients), this study offers robust real-world evidence on PPI safety in SSc, reinforcing PPIs’ vital role in GERD/ILD management while advocating vigilant monitoring for CKD/osteoporosis.* • *Informed Risk–Benefit Clinical Strategy: The findings underscore the need to balance PPI benefits (reducing GERD/aspiration-related lung injury) against amplified renal/bone risks in SSc, guiding individualized treatment and monitoring protocols despite causal limitations of retrospective data.*

**Supplementary Information:**

The online version contains supplementary material available at 10.1007/s10067-025-07686-4.

## Introduction

Systemic sclerosis (SSc) is a chronic autoimmune connective tissue disease characterized by microvasculopathy, immune activation, and progressive fibrosis involving the skin and internal organs [1]. Among its systemic manifestations, gastrointestinal (GI) involvement is a prominent feature [2], with gastroesophageal reflux disease (GERD) affecting 54–90% of patients [3]. This complication often necessitates the use of proton pump inhibitors (PPIs), which remain a cornerstone of treatment for GERD [4]. Beyond improving esophageal symptoms, PPI therapy in SSc has additional benefits, particularly in reducing the risk of aspiration-related lung injury [5, 6]. GERD-induced microaspiration has been implicated as a contributing factor to interstitial lung disease (ILD), a leading cause of mortality in SSc [1]. By mitigating acid reflux, PPIs potentially protect lung function in SSc patients [7, 8].

Despite these benefits, concerns regarding long-term PPI use and associated adverse outcomes have grown. Emerging evidence links prolonged PPI therapy to an increased risk of chronic kidney disease (CKD), osteoporosis, and even neurodegenerative conditions such as Alzheimer’s disease [9–12]. The unique pathophysiology of SSc may amplify the risks associated with PPI use compared to the general GERD population [13]. SSc patients exhibit inherent vulnerabilities—such as microvascular dysfunction, chronic inflammation, and fibrosis—that could synergize with PPI-related adverse effects [2, 3, 7, 9, 13–15]. For example, PPI-induced hypomagnesemia or calcium malabsorption may exacerbate preexisting endothelial injury or osteoporosis, while renal vasculopathy in SSc could potentiate PPI-associated nephrotoxicity [7, 16, 17]. Furthermore, a study by Host et al. demonstrated a significant association between high-dose, long-term PPI use and calcinosis in SSc patients, suggesting a potential link between PPI therapy and mineral metabolism disturbances [7].

This interplay suggests that SSc acts as a “risk amplifier,” compounding the downstream consequences of long-term PPI use beyond what is observed in typical GERD patients.

The potentially heightened risk of CKD in SSc patients undergoing PPI therapy is particularly concerning. CKD is already prevalent in SSc due to renal vasculopathy and secondary complications such as scleroderma renal crisis (SRC) [4, 18]. However, long-term PPI use has been associated with a further increase in CKD risk, potentially through mechanisms such as renal tubular injury or interstitial nephritis [17]. This dual burden raises questions about whether the observed risks of PPI therapy are inherent to the medication itself or reflect underlying disease characteristics in SSc patients.

This study aimed to investigate whether PPIs, commonly used to prevent acid reflux and its effects on the lungs, might increase certain health risks in patients with SSc. The analysis explored the risks of CKD, osteoporosis, vascular dementia and Alzheimer’s disease in patients with SSc compared to the population with GERD and whether the use of PPIs could exacerbate these risks. Understanding this distinction is critical to optimizing PPI use, ensuring that the benefits outweigh the risks in this vulnerable population. This study further highlights the importance of individualized treatment strategies to balance the necessity of PPI therapy with the need for vigilance in monitoring potential side effects.

## Methods

### Study design and data source

For this retrospective observational study, the global, multicenter research network TriNetX was utilized. The platform provides access to data from 102 participating healthcare organizations from 14 countries, encompassing hundreds of medical and research Centers. This network sources de-identified data from billing codes and physician or provider documentation, encompassing a population of over 130 million patients. The analysis, exempt from Institutional Review Board approval, provides a tool for integrated algorithm, that enables cohort selection, comparison of characteristics, and outcomes.

### Study population

Data for analysis spanned from January 1, 2015, to January 3, 2025. Individuals aged 18 years and older were initially identified. From this cohort, those with exposure to PPIs were selected, with exposure defined as having at least three separate documented instances of PPI prescriptions in their medical records. Subsequently, patients with diagnoses of SSc and GERD were identified, requiring at least three separate occurrences of the diagnostic codes in patients’ charts for inclusion. To increase the specificity of diagnostic codes, all patients within the systemic sclerosis group had at least 3 instances of Raynaud’s syndrome documented in their charts. Diagnoses were determined using ICD-10 codes, and medication use was tracked through RxNorm codes. The index event was defined as the first recorded use of a PPI in patients with a pre-existing diagnosis of either SSc or GERD. To maintain distinct cohort definitions, patients with SSc were excluded from the GERD group. Further details on diagnostic criteria and medications can be found in Fig. 1, and Supplementary Material Appendix 1. The study group comprised patients with SSc who used PPIs, while the control group included patients with GERD who used PPIs. It should be noted that the database does not provide information on specific medication doses, frequencies, or routes of administration. No additional exclusion criteria were applied, such as the use of other GERD medications like H2 blockers — groups were matched for the use of these medications. This decision was made to ensure that the study population closely reflected the general population, including individuals who may purchase over-the-counter medications, use them in combination with others, or have additional comorbidities, such as autoimmune conditions.

### Study outcomes

The outcomes of interest were the incidences of overall CKD, CKD stage 3, CKD stage 4, CKD stage 5, End-stage renal disease (ESRD), dependence on renal dialysis, osteoporosis, vascular dementia and Alzheimer’s disease — these were defined by respective ICD-10 codes. Patients with a prior history of any of the aforementioned conditions before the index event were excluded from the outcome analysis.

### Statistical analysis

Baseline patient characteristics were presented as mean ± standard deviation (SD) for continuous variables and compared using independent-sample Student’s *t*-tests. Categorical variables were expressed as n (%) and analyzed using the chi-square test. To ensure group comparability, a 1:1 propensity score matching through the nearest-neighbor algorithm was employed. Matching criteria included age, sex, ethnicity, and comorbidities such as obesity, hypertensive disorders, ischemic heart disease, and diabetes mellitus, along with their associated treatments. Specifically, the matching accounted for exposure to antihypertensive medications (calcium channel blockers, ACE inhibitors, and angiotensin II receptor blockers), antianginals and blood glucose-regulating agents (including GLP-1 receptor agonists and SGLT-2 inhibitors). Given the known association between corticosteroid use and osteoporosis risk, the groups were also matched for exposure to prednisone or methylprednisolone. Patients were also matched for Histamine 2 receptor antagonist use. TrinetX only allows the matching for the frequency of medications present in patient charts, not for doses or routes of administration. Exposure to non-steroidal anti-inflammatory drugs (NSAIDs) was recorded in both groups; however, due to TriNetX’s limitations in differentiating between chronic and intermittent NSAID use—and the commonality of over-the-counter purchases—patients were not matched on this variable. This decision aligns with our overarching aim to ensure that the study populations closely reflect real-world patient characteristics.

While the inclusion of serologic markers such as anti-Scl-70, anti-RNA polymerase III, and anti-centromere antibodies would have enhanced cohort characterization, this information was not available within the TriNetX database.

Balance between the groups was evaluated using standardized mean differences (SMD), with an SMD < 0.1 indicating adequate balance. Outcomes were defined as the presence of at least one relevant diagnostic code in the patients’ medical records. Overall and attributable risks, together with 95% confidence intervals (CIs), and *p*-values were calculated for the outcomes of interest. Statistical significance was determined using two-tailed tests, with a threshold of *p* < 0.05.

## Results

There were 6800 patients in the SSc with PPI group and 1,889,433 in the GERD group. Based on the inclusion criteria, database had 15,278 SSc patients, among these 8478 did not have a history of PPI presence in their charts for more than 3 occasions. Data for inclusion/exclusion and matching criteria were reported by all of the participating healthcare organizations. In both cohorts, the majority of patients were white (68.1% vs 68.5%, SMD 0.08). The age distribution was similar between groups, but the SSc group had a notably higher proportion of female patients (84.1% vs 56.7%, SMD 0.6).

A diagnosis code indicative of progressive disease was documented in 42% of patients with SSc, while codes consistent with CREST syndrome were noted in 48% of cases. It is important to emphasize that these classifications were based solely on the presence of corresponding ICD-10 codes in the patients’ charts, as no standardized clinical definitions for progressive disease or CREST syndrome were available.

Mycophenolate mofetil emerged as the most frequently prescribed immunosuppressant, used in 60% of SSc patients. Both cohorts demonstrated comparable NSAID usage (24% vs. 24%, SMD 0.03). Data on the use of immunosuppressants, vasodilators, and other disease-modifying agents was not consistently reported by all participating healthcare organizations. As a result, the actual usage rates are likely higher than recorded. Please refer to Appendix 1 for the percentage of healthcare organizations that provided medication data. The presence of a single immunosuppressant in a patient’s chart does not rule out the possibility of concurrent use of additional agents.

The GERD group had a higher prevalence of hypertensive diseases (53.9% vs 48.9%, SMD 0.1), obesity or high BMI (25.9% vs 15.4%, SMD 0.26), and diabetes mellitus (24.3% vs 11.9%, SMD 0.32). In contrast, the SSc group demonstrated higher use of calcium channel blockers (54.9% vs 23%) and overall antihypertensive medications (29% vs 15.8%, SMD 0.32). Blood glucose-regulating agents were more commonly prescribed in the GERD group prior to matching (31.5% vs 24.9%, SMD 0.15).

Baseline creatinine levels — levels present on day of index event — were available for 79.1% of patients in the SSc group and 72.7% in the GERD group. The SSc group had higher average baseline creatinine values (1.04 ± 4.26 vs 1.04 ± 1.98, SMD 0.003) and higher prevalence of pulmonary disease **(**Table 1**).** Interstitial lung disease was identified in 33% of patients with SSc. Given this prevalence, we anticipated a higher number of patients receiving Nintedanib or Tocilizumab. However, medication data were not consistently available across all participating healthcare organizations (see Appendix 1). Additionally, it is important to consider the temporal context of our dataset, which spans back to 2015. The use of these therapies likely became more widespread only after key clinical trials—SENSCIS for Nintedanib in 2019 [19], and focuSSced [20] and faSScinate [21] for Tocilizumab, which led to its FDA approval in 2021—were published.

**Table 1:** Baseline patient characteristics of study groups, before and after propensity score matching

Characteristics	SSc + PPI (N = 6800)	GERD + PPI (N = 1,889,433)	Standardized mean difference	SSc + PPI (N = 6790)	GERD + PPI (N = 6790)	Standardized mean difference
Before propensity score matching				After propensity score matching		
Age, mean ± SD	57.4 ± 14.1	58.5 ± 16.6	0.07	57.4 ± 14.1	58.5 ± 15.6	0.08
Female, %	84.1	56.7	0.6	84	83.2	0.02
Hispanic, %	27.2	29.5	0.05	27.2	26.5	0.01
White, %	68.1	68.5	0.08	68.2	69.3	0.02
Black, %	14.3	12.2	0.06	14.4	14	0.008
Asian, %	3.5	3.3	0.01	3.5	3.2	0.01
Unknown, %	14.1	16	0.09	14.2	13.5	0.01
Diagnoses						
Hypertension, %	48.9	53.9	0.08	48.9	50.3	0.03
Ischemic heart diseases, %	19.4	18.9	0.01	19.4	19.9	0.01
Overweight, Obesity, %	15.4	25.9	0.26	15.5	16.2	0.02
Diabetes Mellitus, %	11.9	24.3	0.32	11.9	12.9	0.03
Primary Pulmonary Hypertension, %	14	1		Not matched		
Secondary Pulmonary Hypertension, %	32	3				
Interstitial Pulmonary Disease, %	33	2				
Progressive systemic sclerosis, %	42					
CREST syndrome, %	48					
Medications						
Calcium channel blockers, %	54.9	23	0.7	54.8	57.1	0.05
Prednisone, %	40	22.4	0.4	40.1	39.2	0.02
Methylprednisolone, %	25.5	22.7	0.07	25.6	24.9	0.01
Blood glucose regulation agents, %	24.9	31.5	0.15	24.9	25.9	0.02
Antihypertensives, %	29	15.8	0.32	28.9	29.8	0.02
ACE inhibitors, %	21	22.3	0.03	20.9	20.9	0.004
Antianginals, %	19.3	12.2	0.2	19.2	19.3	0.002
Angiotensin II receptor inhibitors, %	14.7	15.7	0.03	14.7	14.4	0.009
Famotidine, %	19.2	19.2	0.0007	19.2	18.6	0.02
Ranitidine, %	15.8	11	0.14	15.8	16	0.08
Cimetidine, %	1.1	0.7	0.05	1.1	0.9	0.02
Nizatidine, %	0.2	0.2	0.003	0.2	0.2	0.001
Rituximab, %	11.7					
Methotrexate, %	32.2					
Tocilizumab, %	7.2					
Mycophenolate mofetil, %	60			Not matched		
Cyclophosphamide, %	6					
Nintedanib, %	8.8					
Mycophenolic acid, %	20					
Azathioprine, %	17					
Bosentan, %	5					
Iloprost, %	1					
Sildenafil, %	58.8					
Alprostadil, %	1.5					
Non-steroidal anti-inflammatory analgesics, %	24	24	0.03			
Baseline Creatinine availability, %	79.1	72.7	0.003	Not matched		
Baseline Creatinine value	1.04 ± 4.26	1.04 ± 1.98	0.2			

Before matching, the SSc group showed increased risks for chronic kidney disease (CKD) overall, with an attributable risk (AR) of 2.1 (95% CI 1.1–3, *p* < 0.01). This included elevated risks for CKD stages 3 and 4. Risk differences for CKD stage 5, ESRD or dialysis dependence were not statistically significant. Osteoporosis was also more prevalent in the SSc group, with an AR of 11.8 (95% CI 10.8–13, *p* < 0.01). Conversely, the risks of Alzheimer’s disease and vascular dementia were lower in the SSc group.

Post-matching analysis revealed a slightly higher AR for CKD overall in the SSc group (AR 2.8, 95% CI 1.5–4, *p* < 0.01), but minimal to no differences for CKD stages 3, 4, and 5, ESRD, and dialysis dependence. Osteoporosis remained more prevalent in SSc group, while risks of Alzheimer’s and vascular dementia remained higher in the GERD group **(**Table 2**).**

**Table 2:** Risks of kidney disease, Alzheimer’s disease and osteoporosis in patients with systemic sclerosis and PPI exposure versus general population with GERD and PPI exposure

Outcome	Groups	Overall risk, %	Attributable risk, %	Overall risk, %	Attributable risk, %
Before propensity score matching				After propensity score matching	
Chronic kidney disease (CKD)	SSc + PPI	16.5 (960/5808)	2.1 (1.1–3) *P-*value < 0.01	16.5 (959/5800)	2.8 (1.5–4) *P-*value < 0.01
	GERD + PPI	14.4 (231,347/1,601,862)		13.7 (790/5748)	
Chronic kidney disease, stage 3	SSc + PPI	12.4 (783/6295)	1.7 (0.9–2.5) *P-*value < 0.01	12.4 (783/6288)	2.1 (1.05–3.2) *P-*value < 0.01
	GERD + PPI	10.7 (187,231/1,746,924)		10.3 (650/6288)	
Chronic kidney disease, stage 4	SSc + PPI	3.9 (258/6680)	0.9 (0.5–1.4) *P-*value < 0.01	3.9 (257/6672)	0.95 (0.3–1.6) *P-*value < 0.01
	GERD + PPI	2.9 (53,346/1,845,025)		2.9 (192/6607)	
Chronic kidney disease, stage 5	SSc + PPI	1.3 (89/6739)	0.24 (0–0.5) *P-*value 0.06	1.32 (89/6732)	0.05 (−0.3–0.4) *P-*value 0.8
	GERD + PPI	1.08 (20,153/1,865,297)		1.27 (85/6691)	
End-stage renal disease	SSc + PPI	2.8 (184/6574)	0.25 (−0.15–0.6) *P-*value 0.2	2.8 (183/6567)	0.5 (−0.09–0.9) *P-*value 0.1
	GERD + PPI	2.6 (45,827/1,794,393)		2.3 (150/6426)	
Dependence on renal dialysis	SSc + PPI	1.6 (106/6717)	0.26 (− 0.04–0.6) *P-*value 0.06	1.6 (105/6710)	0.1 (− 0.3–0.5) *P-*value 0.5
	GERD + PPI	1.3 (24,456/1,855,983)		1.4 (96/6652)	
Osteoporosis	SSc + PPI	20.6 (1118/5429)	11.8 (10.8–13) *P-*value < 0.01	20.6 (1117/5424)	9 (7.7–10.4) *P-*value < 0.01
	GERD + PPI	8.8 (149,918/1,713,413)		11.6 (687/5936)	
Alzheimer’s disease	SSc + PPI	0.8 (55/6788)	−0.6 (−0.8, −0.3) *P-*value < 0.01	0.8 (55/6,781)	− 0.7 (− 1, − 0.4) *P-*value < 0.01
	GERD + PPI	1.4 (25,590/1,871,770)		1.5 (102/6749)	
Vascular dementia	SSc + PPI	0.5 (34/6789)	− 0.6 (− 0.7, − 0.4) *P-*value < 0.01	0.5 (34/6782)	− 0.6 (− 0.8, − 0.3) *P-*value < 0.01
	GERD + PPI	1 (19,850/1,876,531)		1.05 (71/6760)	

*Notes:* The cohorts were matched with age, race/ethnicity, various diagnoses and medications. Discrepancies between the total number of patients before and after analysis mean that patients were excluded due to the presence of outcomes prior to the index period

The variation in denominators across outcomes in Table 2, even prior to propensity score matching, is primarily due to the exclusion of patients with pre-existing diagnoses of the respective outcomes. For each outcome analysis, patients who had the condition documented before the index event were excluded to ensure temporal clarity and reduce bias. For example, individuals with a prior diagnosis of Alzheimer’s disease were excluded from the Alzheimer’s outcome analysis. This approach was applied consistently across all outcomes and for both the pre- and post-matching cohorts. As a result, discrepancies in patient counts reflect necessary exclusions to maintain the integrity of each outcome-specific analysis.

## Discussion

This retrospective study utilized a large-scale real-world database to compare systemic sclerosis patients using proton pump inhibitors against a control cohort of gastroesophageal reflux disease patients also on PPI therapy. The primary goal of the study was to assess the overall lifetime risks of CKD/ESRD, osteoporosis, vascular dementia, and Alzheimer’s disease in SSc patients on PPI. By focusing on these outcomes, the analysis aimed to assess whether the additional disease burden of SSc compounded the risks associated with PPI use.

The decision to compare GERD patients to SSc patients, rather than SSc patients on PPIs versus those not on PPIs, stemmed from the common use of PPIs in SSc patients. Given the high prevalence of GERD and its pulmonary complications in SSc [1, 12], patients not prescribed PPIs are likely to have milder non-cutaneous manifestations, creating an unfair comparison. By using GERD patients as a control, we aimed to account for shared risk factors while isolating the additional burden posed by SSc.

SSc patients had a lower prevalence of hypertension but higher antihypertensive use, reflecting the frequent use of calcium channel blockers to manage Raynaud’s phenomenon. The SSc cohort also exhibited a high prevalence of pulmonary comorbidities, including interstitial lung disease and pulmonary hypertension. This underscores the importance of avoiding further lung injury in this group, reinforcing the necessity of GERD management with PPIs to reduce aspiration risks [8].

The study revealed that 16.5% of patients with SSc developed CKD following the initiation of PPI therapy, though only a small percentage progressed to CKD stage 4 or higher **(**Table 2). It should be noted, that SSc patients had higher range of baseline creatinine, thus patients who met CKD criteria prior to index event were excluded from the outcome analysis. The findings underscored a heightened risk of CKD in SSc patients compared to the GERD cohort. After propensity score matching, the attributable risk (AR) for CKD was 2.8%, with negligible differences noted in the incidence of advanced CKD stages or dialysis dependence. Given the established link between PPI use and renal disease [10, 22], it is reasonable to assume that PPIs contributes an additive risk to the already elevated baseline susceptibility in SSc patients [17, 18]. The incidence of these conditions appears to follow a similar trend as observed in the GERD group. While there is an elevated risk of CKD in the SSc group, the relatively modest overall difference is unexpectedly reassuring. This is particularly notable given the inherent renal complications of SSc, such as scleroderma renal crisis, which are known to significantly predispose these patients to kidney dysfunction [18]. Notably, NSAID use was comparable between both groups, despite the possibility that patients with systemic sclerosis (SSc) may be more likely to use NSAIDs. While chronicity of use could not be determined, this similarity further reinforces the robustness of the study’s findings.

Osteoporosis was significantly more prevalent in the SSc cohort, with an AR of 9%. This is consistent with prior studies reporting a high prevalence of osteoporosis in SSc, driven by chronic inflammation, malabsorption, and glucocorticoid use [23, 24]. PPI-induced calcium malabsorption may exacerbate this vulnerability, further emphasizing the need for vigilant bone health monitoring in SSc patients [25]. The analysis also revealed that a high percentage of SSc patients had been exposed to steroids.

Conversely, SSc patients on PPI showed a slightly lower risk of Alzheimer’s disease and vascular dementia compared to the GERD group, with an ARs of − 0.7% and − 0.6%, respectively. This may be explained by several factors [26–28],It is possible that unmeasured confounders, such as closer medical supervision in SSc patients or younger age of death may have influenced this result. Also SSc patients frequently receive immunosuppressive therapies (e.g., mycophenolate, rituximab) which may modulate neuroinflammatory pathways implicated in AD pathogenesis, such as microglial activation and cytokine release (IL-6, TNF-α) [29, 30]. Additionally, chronic endothelial dysfunction in SSc may paradoxically upregulate vascular protective factors (e.g., nitric oxide synthase) that mitigate cerebral small vessel disease — a key contributor to vascular dementia [31]. The younger age at SSc diagnosis (mean ~ 57 years in the study cohort) and rigorous monitoring for cardiovascular risk factors may further reduce dementia risk compared to GERD populations with potentially undiagnosed vascular comorbidities.

There is evidence in the medical literature suggesting that hyperuricemia might potentially be protective against Alzheimer’s disease. Multiple meta-analyses and observational studies have found that higher serum uric acid levels are associated with a decreased risk of Alzheimer’s disease, likely due to uric acid’s antioxidant properties [32–34]. Elevated serum uric acid levels are consistently observed in patients with systemic sclerosis and are independently associated with clinical features of microvascular damage, including digital ulcers and worsening peripheral microangiopathy, as demonstrated by nailfold capillaroscopy findings [35]. Higher uric acid levels also correlate with increased intrarenal arterial stiffness and pulmonary hypertension in systemic sclerosis, and are inversely related to functional capacity in affected patients [36, 37]. Thus, it is a possibility that hyperuricemia might also play a protective role against neurodegeneration in SSc patients.

Alternatively, the neuroprotective association could reflect unique PPI effects in SSc. While PPIs are linked to dementia risk in general populations, their acid suppression in SSc may reduce systemic inflammation from chronic GERD, which is associated with IL-1β-driven neuroinflammation via microaspiration [38]. Furthermore, SSc-specific genetic variants (e.g., IRF5, STAT4) [39, 40] may incidentally protect against amyloid-β accumulation or tau hyperphosphorylation. However, these hypotheses require validation through prospective studies incorporating neuroimaging (e.g., MRI for white matter hyperintensities) and biomarker panels (CSF amyloid-β) to account for potential surveillance bias and unmeasured confounders.

It is also important to acknowledge that certain immunosuppressive therapies have been associated with CKD or declining renal function [41]. Given that patients with SSc are more likely to require prolonged exposure to these agents over the course of their disease, this represents a potential confounding factor. However, the relatively modest difference in CKD risk observed in the SSc cohort reinforces the strength and robustness of the study.

This study offers several significant strengths that enhance its clinical and scientific relevance. First, the use of the TriNetX global database provided unprecedented scale, encompassing 6800 SSc patients and 1,889,433 GERD controls—enabling high statistical power to detect even modest risk differences. The real-world design reflects actual clinical practice patterns, increasing the external validity of findings. Second, rigorous 1:1 propensity score matching accounted for 18 demographic, comorbidity, and medication variables (including age, sex, ethnicity, hypertension, diabetes, obesity, antihypertensives, steroids, and H2 blockers), minimizing confounding. Additionally, the analysis of *graded* outcomes (e.g., CKD stages 3–5, ESRD) provided nuanced insights into disease severity, while the exclusion of pre-existing conditions for each outcome ensured temporal association. Finally, the GERD comparator group design mitigated confounding by indication, as contrasting SSc patients against non-SSc GERD patients (rather than SSc non-PPI users) accounted for shared GERD-related risk factors while isolating the additive burden of SSc pathophysiology.

This study’s retrospective design and reliance on real-world administrative data introduce several limitations requiring careful interpretation. Fundamentally, the observational nature precludes causal inferences between PPI exposure and clinical outcomes in systemic sclerosis, as unmeasured confounders (e.g., socioeconomic factors, disease severity gradients) may persist despite propensity score matching. This limitation is compounded by TriNetX platform-specific constraints: (1) Diagnostic and pharmacological misclassification risks from ICD-10/RxNorm code dependencies, particularly for SSc subtyping (limited vs. diffuse) and GERD severity stratification; (2) Absence of serological (anti-Scl-70, anti-centromere) or histopathological data preventing phenotype-specific analyses; Limitation in the subclassification of systemic sclerosis. Definitions for ‘progressive disease’ and ‘CREST syndrome’ were operationalized using ICD-10 codes from patient charts, as standardized clinical definitions were unavailable and (3) Inability to capture over-the-counter PPI use or non-prescription therapies (e.g., calcium supplements), potentially biasing risk estimates. Future prospective studies should implement clinician-adjudicated case validation and integrate patient-reported adherence logs to address these gaps.

A noteworthy limitation centers on exposure definition. The operational threshold for chronic PPI use (≥ 3 prescriptions) lacks granularity to differentiate sustained therapy from intermittent courses, potentially diluting risk estimates for true long-term users. Moreover, the complete absence of dose, frequency, and duration metrics precludes assessment of cumulative exposure effects or dose–response relationships—particularly relevant for outcomes like CKD and osteoporosis where risk escalates with prolonged use [13–15]. These shortcomings could be mitigated in future work through pharmacy dispensation records with Medication Possession Ratio (MPR) calculations and defined daily dose algorithms to quantify actual exposure burden.

Similarly, immunosuppressant data suffer from significant constraints. While we matched for baseline immunosuppressant exposure, the inability to account for cumulative dosing, treatment duration (e.g., mycophenolate years), or synergistic nephrotoxic combinations (e.g., glucocorticoids with calcineurin inhibitors) leaves residual confounding for renal and bone outcomes. For instance, steroid “exposure” metrics may include single-dose administrations alongside chronic regimens, obscuring their contribution to osteoporosis risk. Prospective cohorts should employ time-varying covariate models with detailed medication histories—including indication, duration, and therapeutic drug monitoring—to disentangle PPI effects from background immunosuppression.

Outcome ascertainment presents additional challenges. Binary endpoint definitions (e.g., osteoporosis presence/absence) using billing codes fail to capture subclinical disease progression, such as declining eGFR trajectories or bone mineral density changes.

The lack of serial biomarker data (e.g., urinary albumin-to-creatinine ratios, vitamin D levels) further limits mechanistic insights. Incorporating annual DXA scans with fracture mapping and quarterly renal function panels in longitudinal studies would provide dynamic risk profiling[42, 43].

It is worth acknowledging that some of the outcomes of the analysis might represent inciting events of the others, for example, while scleroderma renal crisis (SRC) could be a driver of CKD, the analysis was not designed to isolate the specific contribution of SRC to the observed outcomes or its potential interaction with PPI therapy. The retrospective, claims-based nature of the TriNetX data lacks granularity to accurately identify specific events and analyze their temporal relationship to PPI exposure. Therefore, while we cannot rule out SRC as a pathway to worsening CKD in some patients, our study provides a holistic assessment of the overall CKD risk associated with PPI use. The relatively small attributable risk suggests that this net effect is modest, offering clinicians valuable reassurance when balancing the essential benefits of PPI therapy against potential renal risks in SSc.

Finally, demographic homogeneity (predominantly White, female cohort) may limit generalizability, while the inability to analyze SSc patients without PPI exposure restricts therapeutic comparisons. Multicenter collaborations targeting underrepresented populations and treatment-naïve subgroups could enhance external validity. Despite these constraints, our pragmatic approach reflects real-world practice patterns, and the large sample size provides clinically relevant insights on risk–benefit tradeoffs—provided results are validated through targeted prospective investigations with the methodological refinements proposed herein.

The small attributable risks observed for CKD and osteoporosis in SSc patients are reassuring, suggesting that while these risks warrant attention, they may not dramatically alter clinical management. Importantly, most SSc patients receive PPI prescriptions from healthcare providers, ensuring closer monitoring compared to over-the-counter use in the general population. This, in turn, reinforces the notion that clinicians may provide some reassurance to patients who express concerns about PPI use.

These findings highlight critical needs for future research: (1) prospective longitudinal studies with serial renal function monitoring (eGFR trajectories, urinary biomarkers), standardized bone health assessments (annual DXA with fracture documentation), and precise PPI exposure tracking (dose-duration validation through pharmacy records); and (2) mechanistic studies of the observed neuroprotective association. Priority should be given to high-risk subgroups — particularly those with existing renal dysfunction or progressive ILD — using time-varying analyses to untangle PPI effects from disease progression and concurrent immunosuppressant toxicity. Such rigorous designs will clarify risk–benefit ratios for PPI therapy in SSc management.

While PPI therapy remains indispensable in managing GERD and protecting lung health in SSc, these findings emphasize the need for vigilant monitoring to mitigate potential adverse effects. This balanced approach ensures optimal patient care without compromising therapeutic benefits.

## Supplementary Information

Below is the link to the electronic supplementary material.

Supplementary file1 (DOCX 132 KB)

## Data Availability

The data utilized in this study were accessed via the TriNetX platform, a federated health research network aggregating deidentified electronic health record data from participating healthcare organizations. Due to institutional data use agreements, privacy regulations, and the federated nature of TriNetX, the raw patient-level data cannot be shared directly by the authors. TriNetX enforces privacy compliance through deidentification and data rounding for small sample sizes, in accordance with HIPAA and other relevant regulations. Researchers interested in replicating or extending the analyses may request access to the TriNetX platform through institutional subscription or collaboration with a participating healthcare organization. Detailed cohort definitions, code lists, and analytic procedures used in this study are available from the authors upon reasonable request, subject to approval by TriNetX and the relevant institutional review boards. The TriNetX platform currently provides access to real-world data from over 150 million electronic health records, supporting robust multicenter research while maintaining strict data privacy and provenance standards. For further information on data access, please refer to TriNetX (https://trinetx.com) or contact the corresponding author.
