# Supplementary material for: Proton pump inhibitors in systemic sclerosis: should we exercise caution? Insights from a large-scale data analysis
Source: Clin Rheumatol. 2025 Sep 12;44(10):4061–70. doi: 10.1007/s10067-025-07686-4 (PMC12518461; doi:10.1007/s10067-025-07686-4)
Supplement: Supplementary file 1 — Supplementary file1 (DOCX 132 KB) [file 10067_2025_7686_MOESM1_ESM.docx]

**Figure 1.** Study Consort Design


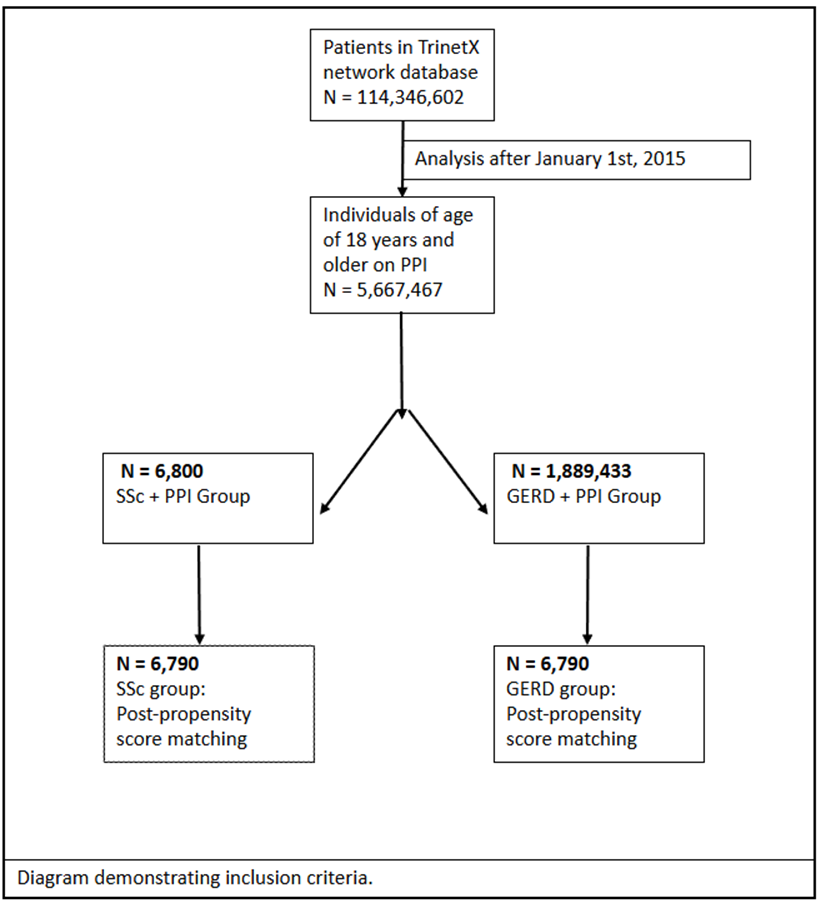


**Appendix 1.** Inclusion/Exclusion Criteria, Matching Criteria and Outcomes with Corresponding ICD-10, VA, RxNorm and TNX Curated Codes

***Inclusion Criteria***

**ICD-10 Codes**

M34 – Systemic sclerosis (SSc) (Contains all diagnoses under the code hierarchy)

K21 – Gastroesphageal reflux disease (GERD) (Contains all diagnoses under the code hierarchy)

**RxNorm Codes for Proton Pump Inhibitors (PPI)**

Esomeprazole (283742) **OR** Dexlansoprazole (816346) **OR** Lansoprazole (17128) **OR** Rabeprazole (114979) **OR** Omeprazole (7646) **OR** Pantoprazole (48790) **OR** Vonoprazan (2604577)

***Matching Criteria***

**ICD-10 Codes**

I10 – I1A – Hypertensive diseases

I20-I25 – ischemic heart diseases

E08-E13 – Diabetes mellitus

E65-E68 – Overweight, obesity and other hyperalimentation

**VA Codes (Encompasses RxNorm Codes within classes) with report rate by the healthcare organizations (HCOs)**

CV200 – Calcium channel blockers

CV490 – Antihypertensives

CV800 – Ace inhibitors

CV250 – Antianginals

CV805 – Angiotensin II inhibitors

HS500 – Blood glucose regulation agents

CN104 – Non-steroidal anti-inflammatory analgesics

**RxNorm Codes**

8640 – Prednisone

6902 – Methylprednisolone

4278 – Famotidine

42319 – Nizatidine

2541 – Cimetidine

9143 – Ranitidine

5640 – Ibuprofen

7258 – Naproxen

41493 – Meloxicam

35827 – Ketorolac

***Other codes for patient characteristics***

**ICD-10 Codes**

I73.0 – Raynaud’s syndrome

I27.0 – Primary pulmonary hypertension

I27.2 – Other secondary pulmonary hypertension

J84 – Other interstitial pulmonary diseases

M34.0 – Progressive systemic sclerosis

M34.1 – CR(E)ST syndrome

**TNX-Curated**

9024 – Creatinine [Mass/volume] in Serum, Plasma or Blood

**Medications and data report rate by the healthcare organizations (HCOs)**

121191 – Rituximab – Reported by 60% of HCOs

6851 – Methotrexate – Reported by 52% of HCOs

612865 – Tocilizumab – Reported by 50% of HCOs

68149 – Mycophenolate mofetil – Reported by 74% of HCOs

3002 – Cyclophosphamide – Reported by 51% of HCOs

1592737 – Nintedanib - Reported by 53% of HCOs

7145 – Mycophenolic acid – Reported by 61% of HCOs

1256 – Azathioprine – Reported by 64% of HCOs

75207 – Bosentan – Reported by 50% of HCOs

40138 – Iloprost – Reported by 10% of HCOs

136411 – Sildenafil – Reported by 72% of HCOs

598 – Alprostadil – Reported by 17% of HCOs

***Outcomes***

**ICD-10 Codes**

N18 – Chronic kidney disease

N18.3 – Chronic kidney disease, stage 3 (moderate)

N18.4 – Chronic kidney disease, stage 4 (severe)

N18.5 – Chronic kidney disease, stage 5

N18.6 – End stage renal disease

M81 **OR** M80 – Osteoporosis with **OR** without current pathological fracture

G30 – Alzheimer's disease

F01 – Vascular dementia
